# Treatment of Complex Femoro-popliteal Lesions: Time to Revise the Guidelines According to Clinical Reality

**DOI:** 10.1007/s00270-023-03589-6

**Published:** 2023-11-07

**Authors:** Thomas Zeller

**Affiliations:** Department Angiology, Clinic for Cardiology and Angiology, University Heart Center Freiburg-Bad Krozingen, Südring 15, 79189 Bad Krozingen, Germany

The prevalence of peripheral arterial disease (PAD) is steadily increasing, affecting over 202 million individuals worldwide [1] with the culprit lesion predominantly located in the femoro-popliteal vessel segment, in particular in patients suffering from claudication [2]. In the past decade, emerging endovascular technologies including drug-coated balloons [3, 4], drug-eluting stents (DES) [5, 6], endoprostheses [7, 8], and atherectomy devices [9] resulted in significant improvements of acute treatment success and durability of endoluminal femoro-popliteal interventions. Nevertheless, international guidelines still consider femoro-popliteal lesions longer than 25 cm as primary bypass indication [2]. However, when the latest guidelines were published like the ESC/ESVS guidelines in 2018, results of two major prospective randomized controlled trials comparing bypass surgery with up-to-date interventional techniques in TASC II C & D femoro-popliteal lesions like the SuperB trial [10] and the ZILVERPASS trial [11, 12] were not yet published.

The SuperB (SUrgical versus PERcutaneous Bypass) study compared the implantation of ePTFE-covered nitinol stent (Viabahn, W.L. Gore, Flagstaff, AZ, USA) with intended autologous venous bypass graft insertion. One-hundred twenty-nine patients were randomized of which 125 patients were treated": 63 in the endoluminal and 62 in the surgical group. Patients were treated for critical limb-threatening ischemia (CLTI) in 38.1% and 32.2% and in the endoluminal and surgical arm, respectively. Mean lesion length was 23 cm in both groups, and the majority of lesions were TASC II D lesions. Thirty-day morbidity was significantly lower in the endoluminal group, as was hospitalization time. There were no significant differences in Rutherford category between groups at any time point. At 30 days, patients in the endoluminal group showed a greater improvement in QoL scores not persisting until 1 year. At 1 year, there were no differences in primary, assisted-primary and secondary patency and target lesion revascularization (TLR) rates between groups. Limb salvage rate was 100% in both groups [10].

To date, besides BASIL-1 [13] the ZILVERPASS (ZILVER PTX stent versus prosthetic above-the-knee bypass surgery in femoropopliteal lesions) study is the only RCT with 5-year outcome published data [14]. The study included 220 patients, 113 patients randomized to ZILVER PTX DES (Cook Medical, Bloomington, IN, USA) and 107 patients randomized to above-the-knee prosthetic bypass group. Overall mean lesion length was 247 mm and did not differ between the groups. At one, three and five years, no significant difference in primary patency and TLR rate was found (primary patency: 74.4%, 53.3% and 49.3%, respectively for the ZILVER PTX group versus 72.4%, 57.3% and 40.7%, respectively for the bypass group, freedom from TLR: 80.8%, 66% and 63.8% for the ZILVER PTX group versus 76.1%, 65.9% and 52.8% for the bypass group). Freedom from amputation rate at 5 years was 94.6% in the ZILVER PTX group versus 92.5% in the bypass group (*p* = 0.5818) without a difference when performing a sub-analysis for claudicants versus CLTI patients. At 5 years, no significant difference in survival rate could be seen (ZILVER PTX 69.1% vs. bypass group 71%).

In the past, the ZILVERPASS study with criticized for using prosthetic bypass graft material for the surgical study cohort. Even if the international guidelines recommend the use of autologous venous bypass material, study results are sparse supporting this recommendation at least for above-the-knee bypass insertion [15, 16]. Moreover, as shown in the BEST-CLI trial (surgery or endovascular therapy for chronic limb-threatening ischemia), in patients with advanced atherosclerotic disease adequate great saphenous vein (GSV) is not available in a considerable number of patients, 21.6% of randomized patients in the BEST-CLI study lacked an adequate GSV [17]. It is important to note that some of the study investigators in the ZILVERPASS trial noted that prosthetic bypass was the standard of care for above-the-knee lesions at their medical facility.

The conclusion of both recent RCTs is similar clinical outcomes in a mainly claudicant patient population suffering from complex femoro-popliteal disease regarding survival, freedom from TLR and vessel patency up to 5 years for up-to-date endoluminal revascularization and above-the-knee bypass surgery regardless of the bypass material used. Besides this, an economic analysis of the ZILVERPASS study at three years demonstrated a clear cost–benefit in favor of the endoluminal approach for Germany and the US from the payer’s perspective [12].

As a consequence, according to these two RCTs and clinical reality, guidelines should be adjusted recommending both revascularization strategies as being equally effective.

What is clinical reality? Even if in some countries certified vascular centers are established providing both revascularization options, endoluminal and surgical, it is rare that both revascularization techniques can be offered in a similar quality 24 h/7 days a week. Therefore, not only a patient and lesion characteristics tailored individual treatment decision should be considered but if the best possible treatment can be offered in given center. If not, referring an individual patient to another center of excellence should be considered. For this purpose, vascular center networks may be worth to be established.
